# Beyond Ease of Use: Dynamics of Technology Adoption and Cognitive Load in AI-Assisted Programming for Non-Technical Students

**DOI:** 10.3390/bs16081405

**Published:** 2026-08-17

**Authors:** Rafael Mellado, Claudio Cubillos, Silvana Roncagliolo

**Affiliations:** 1 Escuela de Comercio, Pontificia Universidad Católica de Valparaíso, Valparaíso 2340025, Chile; rafael.mellado@pucv.cl; 2 Escuela de Ingeniería Informática, Pontificia Universidad Católica de Valparaíso, Valparaíso 2362807, Chile; silvana.roncagliolo@pucv.cl

**Keywords:** generative artificial intelligence, technology acceptance model, cognitive friction, non-STEM students, intrinsic motivation

## Abstract

The integration of Generative AI (GenAI) into non-STEM education introduces cognitive demands that challenge static interpretations of technology acceptance. Evidence on GenAI acceptance is drawn largely from cross-sectional designs applied to general-purpose academic tasks and for computing students who already hold prior technical knowledge, leaving unresolved how acceptance perceptions evolve when non-technical students confront sustained high-load programming work. To address this gap, a pre-test/post-test randomized field experiment with repeated measures was conducted with 117 Chilean Accounting and Auditing undergraduates assigned to a control group (instructional videos; n = 57) or an experimental group (Google Gemini 1.5; n = 60). Participants completed Java programming exercises that increased in cognitive complexity, ranging from basic conditionals to vector operations. The study did not assess objective learning outcomes; instead, it tracked changes in user perceptions through a hybrid motivational framework integrating Perceived Ease of Use and Behavioral Intention with Perceived Enjoyment and Value/Usefulness. Baseline results showed lower initial Perceived Ease of Use in the GenAI group, consistent with initial cognitive friction. Post-intervention analyses indicated that the control group showed a significant increase only in Behavioral Intention, whereas the experimental group showed statistically significant within-group gains across all measured dimensions. These findings suggest that, for non-STEM students, the perceived ease and value of GenAI are not necessarily immediate but may develop through sustained interaction with cognitively demanding tasks. The study contributes to technology acceptance research by framing ease of use as an acquired proficiency in high-friction GenAI learning environments.

## 1. Introduction

The digital transformation of the business sector has required non-STEM professionals to move from passive users to active evaluators of algorithmic logic. This transition is hindered by the substantial cognitive load associated with traditional programming languages such as Java, often leading to early academic frustration and technology rejection among business students. Computational thinking has evolved from a purely technical skill to a broader competency centered on problem understanding rather than rote syntax memorization. Despite this evolution, integrating programming into non-STEM curricula remains difficult. Many students perceive the cognitive demands of languages such as Java, C#, or Python as significant obstacles. Previous efforts have primarily utilized passive, asynchronous resources, particularly instructional videos. Although these resources are accessible, they are inherently static and do not provide adaptive, real-time support when students encounter challenges with complex programming abstractions.

Generative Artificial Intelligence (GenAI) is described as enabling a shift toward ‘natural language programming’ (Bray, 2024), positioning AI as an interactive support tool. However, adopting GenAI incurs adjustment costs. Unlike passive resources such as instructional videos, GenAI introduces initial cognitive friction, requiring a period of technical and conceptual adaptation. This process challenges the traditional assumptions of the Technology Acceptance Model (TAM), which generally treats ease of use as a static baseline rather than as a proficiency to be acquired.

Three features of the current evidence on GenAI acceptance limit its relevance to the population and tasks examined here. First, this evidence rests predominantly on cross-sectional designs in which acceptance is measured once and in connection with general-purpose academic activities such as retrieval, drafting, or summarizing, so that operational ease is presumed rather than observed under cognitive strain (Strzelecki, 2024; Vhatkar et al., 2024). Second, studies situating GenAI within programming instruction typically recruit computing or engineering students whose prior exposure to formal syntax lowers the entry cost of the task, leaving learners without that preparation undocumented (Avouris et al., 2025; Feng et al., 2024). Third, TAM and its extensions position Perceived Ease of Use as a comparatively stable antecedent of behavioral intention (Granić, 2023; Or, 2024), an assumption that repeated measurement has rarely tested where the tool itself imposes a learning cost alongside the subject matter, although extended usage periods have been examined for established learning technologies (Sprenger & Schwaninger, 2021). The resulting deficit concerns the temporal behavior of acceptance under high cognitive load among students for whom the programming task and the conversational interface are simultaneously unfamiliar, which this study addresses through pre- and post-intervention measurements across five weeks with participants whose curriculum contains no prior formal programming.

This study investigates the dynamics of technological acceptance and the adoption of GenAI as a scaffolding tool in non-STEM programming education. By comparing GenAI with traditional video-based instruction, the research shifts focus from classical utilitarian acceptance models to a motivational-adoption framework. The analysis centers on whether conversational agents influence the development of intrinsic motivation (Value/Usefulness, Perceived Enjoyment) and technical friction (Perceived Ease of Use) in ways that affect Behavioral Intention. The study also examines how varying levels of task complexity, from low to high cognitive load, influence technology use among students with non-technical backgrounds. The following research questions are proposed:

1. RQ1: How does the integration of GenAI as a personalized tutor, compared to passive video instruction, alter the evolutionary trajectory of technical friction (Ease of Use) and intrinsic motivation (Value/Usefulness, Perceived Enjoyment) to shape behavioral intention among non-STEM students?
2. RQ2: How do students navigate the “initial cognitive friction” introduced by GenAI, and to what extent does overcoming this technical barrier affect their final behavioral intention to adopt the technology?
3. RQ3: In what ways does the increasing cognitive load of the programming tasks (from basic conditionals to complex arrays) contextualize the perceived enjoyment and the behavioral intention to continue using GenAI in populations lacking foundational technical skills?

The study is situated in a geographical and educational context that remains underrepresented in high-impact research, and its participants, unlike STEM students in high-income systems, frequently enter the course without consolidated logical-mathematical preparation, so that the cognitive demands of programming constitute a substantial obstacle to their learning. Under such conditions, the adoption of GenAI extends beyond efficiency and may operate as a motivational resource that sustains engagement with tasks that students would otherwise avoid. Collecting evidence in this setting responds to Andrade-Girón et al. (2024), who emphasize the need for Latin American data to broaden the evidence base concentrated in Anglo-American contexts and examine disruptive tools outside elite technological ecosystems.

The principal theoretical contribution of this study is the shift from traditional utilitarian adoption models to an extended motivational framework applicable to contexts of high cognitive friction. Instead of conceptualizing Perceived Ease of Use as a static precursor to adoption, the research frames it as an acquired proficiency that develops after students overcome the dual challenges of programming logic and GenAI’s initial learning curve. The observed concurrent increases in intrinsic motivation (Value/Usefulness and Perceived Enjoyment) and final behavioral intention following initial technical friction provide an expanded behavioral perspective on how non-experts adopt disruptive, high-load cognitive tools.

The remainder of the article is organized as follows: Section 2 presents a literature review that integrates the importance of programming in non-STEM disciplines, the state of the art of GenAI in education, and the theoretical foundations of the TAM. Section 3 details the methodology, including the experimental design, sample, and data-collection instruments. Section 4 presents the quantitative results and the analysis of intrasubject variance (RCI). Section 5 discusses the findings, considers existing theory, addresses pedagogical relevance, and outlines the study’s limitations. Finally, Section 6 presents the conclusions and directions for future research.

## 2. Literature Review

### 2.1. Importance of Programming in Non-STEM Curricula

The integration of programming into higher education now extends beyond engineering disciplines and is treated as a competency of general professional relevance, which displaces the emphasis from coding as a technical operation toward computational thinking as a cognitive skill that allows students in non-STEM fields to formulate problems and design solutions rather than memorize syntax (Gómez & Suarez, 2023; Kim, 2023). Within non-scientific disciplines, this competency updates graduate profiles, supports advanced problem-solving, and is increasingly incorporated into the curriculum rather than being offered as an isolated module (Rojas-López & García-Peñalvo, 2020), although the magnitude of its effect on individual skill acquisition still requires empirical assessment (Díaz & Silvain, 2020).

Teaching practice is adapting to no-code and low-code platforms and AI-driven tools, which rely on visual elements and natural language to lower technical barriers and allow students to concentrate on design principles and solution strategies rather than manual coding (Kim, 2023). This shift toward logical understanding over syntactic output has prompted debate about the necessity of teaching traditional algorithms such as sorting, which can now be generated from natural language prompts (Denny et al., 2024; Kim, 2023), and places AI literacy and abstraction among the constitutive components of contemporary computing instruction (Avouris et al., 2025; Martini, 2024; Rubio-Manzano et al., 2025).

The automation of code generation does not relieve educators of their responsibility for conceptual understanding, and it raises the value of reading, debugging, evaluating, and refining generated output, which is now at least as consequential as writing code manually (Andrade, 2023; Prather et al., 2023). Assessment strategies are correspondingly expected to prioritize contextual comprehension and optimization over code production (Peñalvo et al., 2024), and a hybrid arrangement combining human judgment with AI assistance is advocated so that students treat these systems as support while remaining aware of their limitations and the integrity risks associated with excessive reliance (Beale, 2025; Denny et al., 2023; Terrón, 2024). Therefore, debugging and algorithmic thinking retain their pedagogical function, since without them generative systems operate as opaque instruments that the student cannot interrogate (Dickey et al., 2024; Duarte, 2023; Feng et al., 2024).

### 2.2. Generative Artificial Intelligence as a Cognitive Tool

#### 2.2.1. GenAI Adoption Dynamics in Higher Education

The incorporation of GenAI into higher education is now described less as a technological novelty than as a component of a broader digital transformation, and Machado et al. (2025) characterized its adoption as an irreversible trend with consequences for research cycles, teaching practice, and scientific communication, which displaces institutional positions from restriction toward regulated incorporation.

The benefit most frequently attributed to these systems is the personalization of learning, since advanced models support virtual laboratories and individualized learning paths and generate content adjusted to the student (Radhwan & Radhwan, 2001; Tariq & Tariq, 2001), while from an operational standpoint, students recognize savings in time and improved management of their work (Andrade-Girón et al., 2024), and instructors report simplified administrative processes, subject to verification of the accuracy of what the system produces (Zheng et al., 2025).

Institutional responses remain fragmented, as Hashmi and Bal (2024) note the absence of coherent implementation strategies and call for transparency and accountability, while comparative reviews of university guidelines reveal divergent orientations, as documents from 116 institutions in the United States concentrate on writing and leave gaps in STEM and coding (McDonald et al., 2024), whereas among 67 German institutions, a majority judge that the benefits outweigh the risks (Christ-Brendemühl, 2025).

The literature characterizes GenAI as a double-edged instrument, associated with higher participation and improved outcomes but also with concerns about academic integrity, equity, and the digital divide (Francis et al., 2025; Izquierdo-Álvarez et al., 2001), with pressure on assessment models whose validity flexible generation may undermine (Batista et al., 2024), and with psychosocial effects, including comparison over technological skill and overreliance that may erode critical thinking (Vieira & Mesquita, 2025).

Responses to these risks displace attention from the tool toward the competencies required for its use. Deroncele-Acosta et al. (2025) identify 15 transversal skills, including critical thinking, ethics, and leadership, as conditions of sustainable adoption, and García (2023) proposes a hybrid human–AI arrangement in which the system provides generative and evaluative support while creativity and critical judgment remain human functions.

#### 2.2.2. GenAI and Cognitive Scaffolding in Programming Education

Research on programming instruction describes a displacement from manual coding toward natural language formulation and logical supervision. Bibliometric analyses report rapid growth in this literature, along with a shift in attention from technical tooling to pedagogical concerns (Ng & Ho, 2025; Vhatkar et al., 2024), and Prather et al. (2023) argue that language models require curricular revision that privileges conceptual understanding over syntax issues.

Instructional approaches that were previously unavailable have become feasible, as Bray (2024) documents that allowing students to describe data transformations in ordinary language raises engagement and performance by reducing the cost of syntax, and Rubio-Manzano et al. (2025) advocate AI-generated visualizations and simulations that direct attention toward execution and debugging rather than composition from scratch. Guo et al. (2025) found that GenAI-assisted project-based learning supports creativity and algorithmic thinking, while cautioning that without deliberate instructional design it does not necessarily improve critical thinking or collaboration.

Expert supervision remains necessary, as large language models handle complex Java tasks yet require continuous human oversight to sustain code quality and correct execution (Tosi, 2024), follow standard analytical procedures only partially, which makes conceptual understanding a condition of safe use (Schwarz, 2025), and their ethical incorporation is constrained by limited training among supervisors and by unresolved questions of data privacy and bias (Poornesh, 2024; Sehmi et al., 2025).

Student engagement with these tools follows two opposing patterns, since improvements in motivation, self-efficacy, and autonomous problem-solving have been reported by students who value feedback they describe as specific and corrective (Hernandez et al., 2025; H.-J. Li et al., 2025), whereas Rahe and Maalej (2025) identified learners who proceed by trial and error and request complete solutions without examining the code they receive, a response consistent with cognitive friction, since students overwhelmed by the demands of the prompt-response cycle complete the task superficially instead of engaging with the underlying concepts, at the cost of their long-term autonomy. Zastudil et al. (2023) added that students and instructors diverge in their judgments about when and how these tools should be used.

### 2.3. Technological Acceptance

#### 2.3.1. The Technology Acceptance Model and Its Application in Educational Settings

The Technology Acceptance Model remains the dominant framework for explaining the adoption of information systems, and it attributes usage decisions primarily to two evaluations formed by the user, namely Perceived Usefulness and Perceived Ease of Use. Although the model originated in organizational research, its constructs have been transferred to management, tourism, healthcare, and education, and Musa et al. (2024) documented through bibliometric analysis that this diffusion continues to expand into digital consumer markets. Its portability is not unconditional, since Silva and Silva (2001) caution that usefulness and ease are interpreted through cultural expectations rather than as universal categories, while Taherdoost et al. (2024) and Chabani and Askri (2001) argue that the model must be integrated with individual, organizational, and social determinants to remain informative under disruptive innovation.

In educational settings, the model is treated as valid but incomplete. Granić and Marangunić (2019) confirm in a systematic review that TAM predicts the adoption of learning technologies with reasonable accuracy, while noting that its explanatory power declines when external variables are omitted from the model. Zaineldeen et al. (2020) extend this criticism by observing that the model overlooks peer influence and institutional support when applied in isolation. Granić (2023) identifies a further limitation in the scarcity of post-adoption theory capable of describing what happens to acceptance once a technology has been used over time. Or (2024) sharpens the practical implication, since the meta-analysis reports that Perceived Ease of Use exerts a direct effect on actual use, independent of behavioral intention, which places the removal of technical barriers on the same plane as motivational intervention.

Two lines of evidence indicate that acceptance is not fixed at the time of first contact. Tao et al. (2022) provided longitudinal data showing that acceptance and usage co-evolve, with growing familiarity improving the initial evaluation of the system, whereas Sprenger and Schwaninger (2021) observed the opposite trajectory for mobile virtual reality, whose perceived usefulness declined markedly after three months of use, whereas classroom response systems retained their acceptance. Arthur et al. (2023) documented a related asymmetry, since student attitudes deteriorate when a device is experienced as an additional effort rather than as support. Bali et al. (2025) confirmed that usefulness and ease remain the principal predictors of behavioral intention in mobile language learning. These results indicate that a single construct may rise or fall according to the demands the technology places on the learner, which is the condition examined in this study.

The model also accounts for rejection, and this reverse reading is pertinent to environments in which the tool imposes an entry cost. Kirlidog and Kaynak (2011) proposed inverting the constructs and treating the Perceived Difficulty of Use and Perceived Uselessness as the operative determinants of non-adoption, an approach that reframes friction as a measurable component of the acceptance process rather than as noise in the measurement of ease.

#### 2.3.2. Acceptance of GenAI Tools

The expansion of GenAI has renewed interest in TAM while exposing the limits of its original constructs, since Perceived Usefulness and Perceived Ease of Use continue to predict adoption without fully accounting for the uptake of non-deterministic agents. Extended formulations in which system quality and information quality feed perceived usefulness, which in turn determines intention, have been supported across academic and professional populations (Almeida et al., 2025; Duong et al., 2023; Ibrahim et al., 2025; Wang et al., 2026). However, Sukirman et al. (2024) reported that students who judged ChatGPT as both useful and easy to use did not necessarily use it frequently, which indicates a distance between perceived capability and effective use, which attitude, rather than ease, appears to mediate.

The opacity of generative systems introduces determinants that utilitarian software does not require. Explainability, ethical perception, and perceived risk have been identified as conditions of adoption (Garcia et al., 2023; Jo, 2025; Ursavaş et al., 2025), and K. C. Li et al. (2025) reported that anxiety toward artificial intelligence discourages use, while self-efficacy and favorable attitudes encourage it, a pattern consistent with the role that Arpaci (2021) attributes to security and privacy concerns in the adoption of the cloud technologies on which current GenAI services rest.

The hedonic and creative dimensions of the interaction constitute a further departure from utilitarian software, since Gao et al. (2024) found that in generative contexts perceived enjoyment frequently displaces usefulness as the mechanism through which users overcome initial operational barriers, Deng et al. (2025) identified personal innovativeness as a disposition that accelerates the adoption of AI-generated content, and Greiner et al. (2023) positioned these tools as a means of reducing cognitive overload in decision-making. Evaluating adoption in demanding tasks therefore requires intrinsic motivation constructs alongside utilitarian ones.

Adoption also diverges by academic role, since student uptake responds to peer influence and collective norms (Kanont et al., 2024), whereas faculty uptake depends on institutional trust, professional development, and access to tools that preserve academic integrity (Ahmed, 2024; Robinson, 2025; Shata & Hartley, 2025), and Nevárez Montes and Elizondo-Garcia (2025) report demographic patterns in Mexico and Spain that run contrary to assumptions about digital nativity.

Although this body of work documents a wide range of predictors, including digital familiarity (Thongkoo et al., 2020; Yeni & van der Meulen, 2022), task-technology fit (Rafi et al., 2024), and anticipated pedagogical utility (Liu et al., 2025), it treats Perceived Ease of Use as a baseline condition established in low-complexity environments. The framework adopted here proceeds from the opposite premise, namely that the opacity of GenAI and the unfamiliarity of prompt formulation generate pronounced initial friction in high-load tasks, so that ease of use is better understood as an acquired proficiency, and the intrinsic motivation constructs of Perceived Enjoyment and pedagogical value/usefulness operate as the mechanisms through which students absorb that friction and sustain behavioral intention.

This study is part of a broader research program examining the role of generative artificial intelligence in programming education. In Mellado and Cubillos (2025), Microsoft Copilot (GPT-4 Turbo) was compared with instructional videos among 71 third-year Industrial Engineering students learning PHP/web programming, using objective knowledge tests and HMSAM-based measures of cognitive absorption and technology adoption. In addition, Cubillos et al. (2025) examined Google Gemini 1.5 and educational videos among 40 Computer Engineering students in a Data Structures course taught in C, focusing on learning outcomes, intrinsic motivation, and perceptions of the learning environment. In contrast, the present study analyzes a sample of 117 Accounting and Auditing students enrolled in a Digital Literacy course, addresses Java programming tasks, and focuses specifically on the evolution of technology acceptance and motivational perceptions under conditions of cognitive friction.

## 3. Method

For the present study, a pre-test/post-test randomized field experiment with repeated measures was adopted, following a quantitative research methodology based on the approach of Mellado and Cubillos (2024). This specific nomenclature reflects the execution of automated random assignment within a naturalistic educational setting, ensuring high ecological validity while maintaining experimental control. A pre-test–intervention–post-test scheme was implemented, with results measured at the pre-test and post-test stages using the TAM questionnaire.

Google Gemini, powered by the Gemini 1.5 model, was selected as the GenAI tool because it is available at no cost to students through university-provided Google Workspace for Education accounts and offers real-time content generation consistent with the objective of teaching Java for data analysis. Other tools accessible through the same institutional accounts, such as ChatGPT or Microsoft Copilot, may offer more accessible interfaces or education-specific features, but students were already familiar with Gemini from previous course activities.

### 3.1. Participants

This study involved second-year students majoring in Accounting and Auditing at a Chilean university. A total of 117 students aged between 18 and 23 years participated in the intervention, which was conducted during the second semester of 2024 and the first semester of 2025.

The individual student was the unit of randomization. Simple random assignment was performed automatically by the algorithmic group allocation function of the Moodle Learning Management System prior to the baseline measurement, without manual intervention from the instructional team, so that each student had an equal probability of being assigned to either condition. Moodle was configured to conceal group composition, and each participant could view and interact exclusively with the materials of the assigned condition, preventing cross-contamination. Participation was voluntary, and students who did not complete the five-week cycle were excluded from the analysis, which accounts for the numerical imbalance between the final groups of 57 in the control condition and 60 in the experimental condition.

Block randomization on demographic covariates or prior programming experience was not implemented, given the logistical constraints of an authentic classroom setting. Nevertheless, the risk of selection bias is limited by the curricular homogeneity of the sample, since all participants shared the same business-oriented academic profile and were encountering object-oriented programming for the first time in a formal academic context.

Baseline contrasts were computed on the pre-test scores to examine pre-existing differences in the acceptance constructs. No significant differences between the groups were observed for enjoyment (Student’s *t*-test, *p* = 0.935) or Behavioral Intention (Student’s *t*-test, *p* = 0.489). A significant difference was obtained for Perceived Ease of Use (Wilcoxon rank-sum test, W = 411.5, *p* < 0.001), with lower initial scores in the experimental condition. As shown in the results, the experimental condition also began from a lower descriptive level in terms of value/usefulness. Therefore, random assignment balanced the groups on the affective constructs but not on the two constructs most closely tied to the tool’s operational demands, and this baseline asymmetry is considered in the interpretation of the results and in the limitations.

### 3.2. Curriculum

The experimental activity was conducted as part of the “Digital Literacy” course in the third-semester Accounting and Auditing curriculum. The course aims to develop computational thinking in non-technical students and is structured into five progressive modules: foundations of algorithmic logic; introduction to the Java language (variables and syntax); flow control structures (conditional statements and loops); static data structures (vectors and matrices); and automation applications for financial data analysis. The course adopts a theoretical–practical approach (learning by doing), combining lecture sessions with programming laboratories focused on business problem-solving.

For this study, the intervention focused on two specific learning objectives (LO), selected to represent different levels of cognitive complexity and technical abstraction:

1. LO1: Apply logical decision structures (if-else and switch statements) to model simple business rules in Java, ensuring the correct execution of the control flow.
2. LO2: Implement and manipulate one-dimensional data structures (vectors) for the storage and bulk processing of information, applying traversal and search algorithms to resolve practical data management cases.

The difference in complexity between the two objectives follows the hierarchy of cognitive abstraction established in programming didactics. LO1 was treated as having low complexity because selection statements maintain a direct semantic correspondence with everyday decision-making, allowing novice students to draw on pre-existing mental models and reducing intrinsic cognitive load (Mayer, 2013). LO2 is treated as high complexity because the manipulation of vectors requires the student to dissociate the index from the value and to hold the state of multiple elements simultaneously (Fincher & Robins, 2019), a demand that, combined with the iteration required for traversal, saturates the working memory and raises technical friction beyond verbal logic.

### 3.3. Process

**Figure 1:**
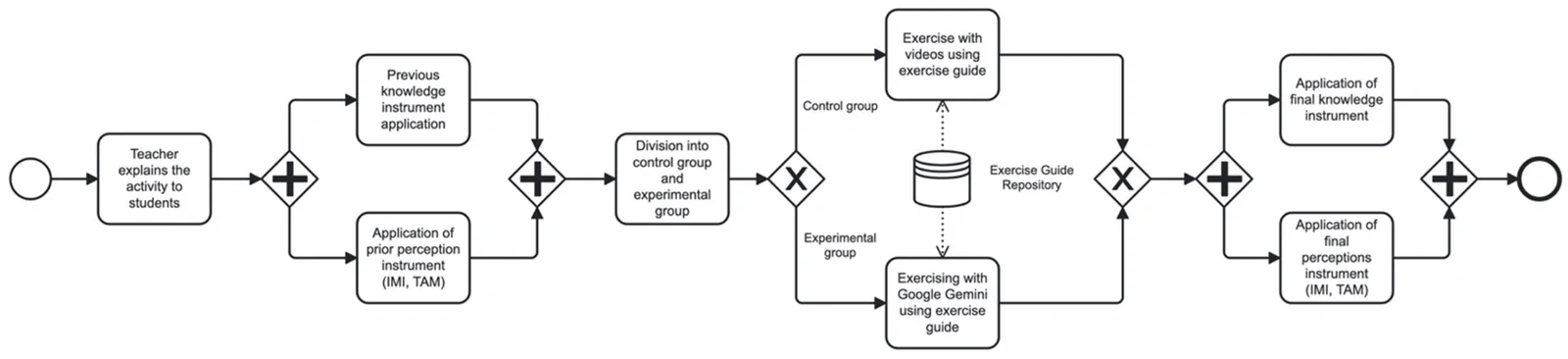
Experimental process used.

Figure 1 outlines the methodological flow of the intervention, which is organized into four sequential stages, namely instructional framing, baseline measurement, differentiated intervention, and output measurement. The design combined an initial synchronous phase with an extended period of autonomous practical work to preserve a realistic learning environment.

Stage 1 consisted of a single joint session for all students, in which the teaching team presented the learning objectives and detailed the structure of the practical activity without revealing the study hypotheses. Students then had a continuous five-week window to complete the remaining stages remotely and at their own pace. In Stage 2, a diagnostic evaluation established the baselines for the acceptance and motivation constructs, integrating dimensions from the Intrinsic Motivation Inventory for enjoyment and the Technology Acceptance Model for ease of use and behavioral intention. Stage 3 was the core of the intervention. Participants tackled the same set of programming exercises, covering topics from conditionals to vectors, hosted in the central course repository, and managed their study time as they saw fit, but under different scaffolding modalities, since the control group relied exclusively on asynchronous video capsules, while the experimental group used Google Gemini as an interactive tutor. This segregation isolates the effect of the conversational agent from that of a passive and highly familiar resource on the user. In Stage 4, upon completing the exercises within the five-week deadline, students completed the post-test perception questionnaire. No training on prompt engineering was provided before the intervention, a decision that, combined with five weeks of autonomous work, allowed participants to interact freely with the tool and reproduced a prolonged adoption scenario without standardization protocols proper to a controlled laboratory.

**Figure 2:**
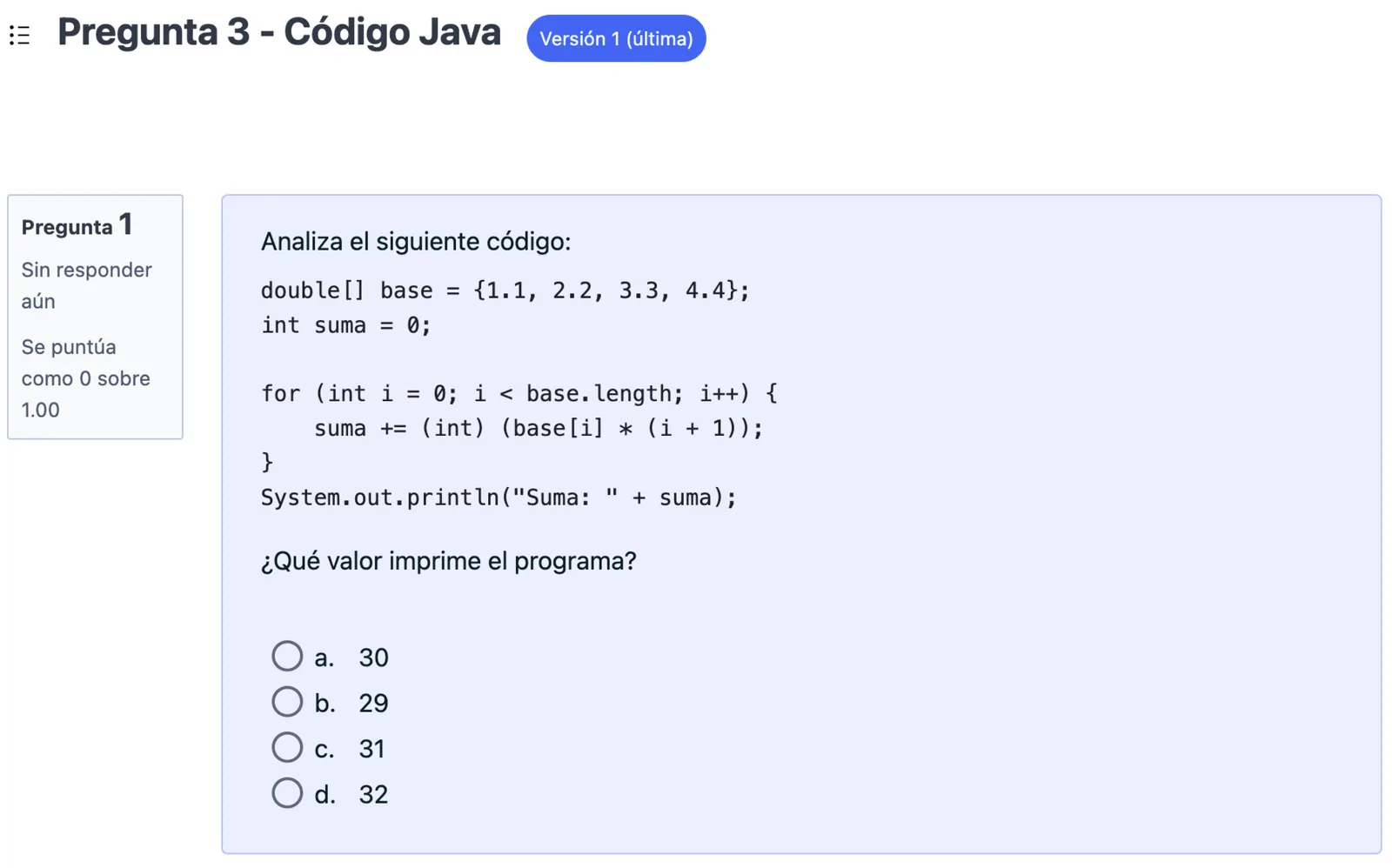
Example of a question used in the practice questionnaire.

Both groups used the same practice guide, consisting of multiple-choice questions drawn from a repository of 108 items developed by the instructor and aligned with LO1 and LO2. Moodle randomly selected ten questions per attempt, with unlimited attempts. Figure 2 shows an example item that requires a Java program to store the grades of ten students in a vector and compute the group average, thereby combining the data structure of LO2 with the control flow of LO1.

**Figure 3:**
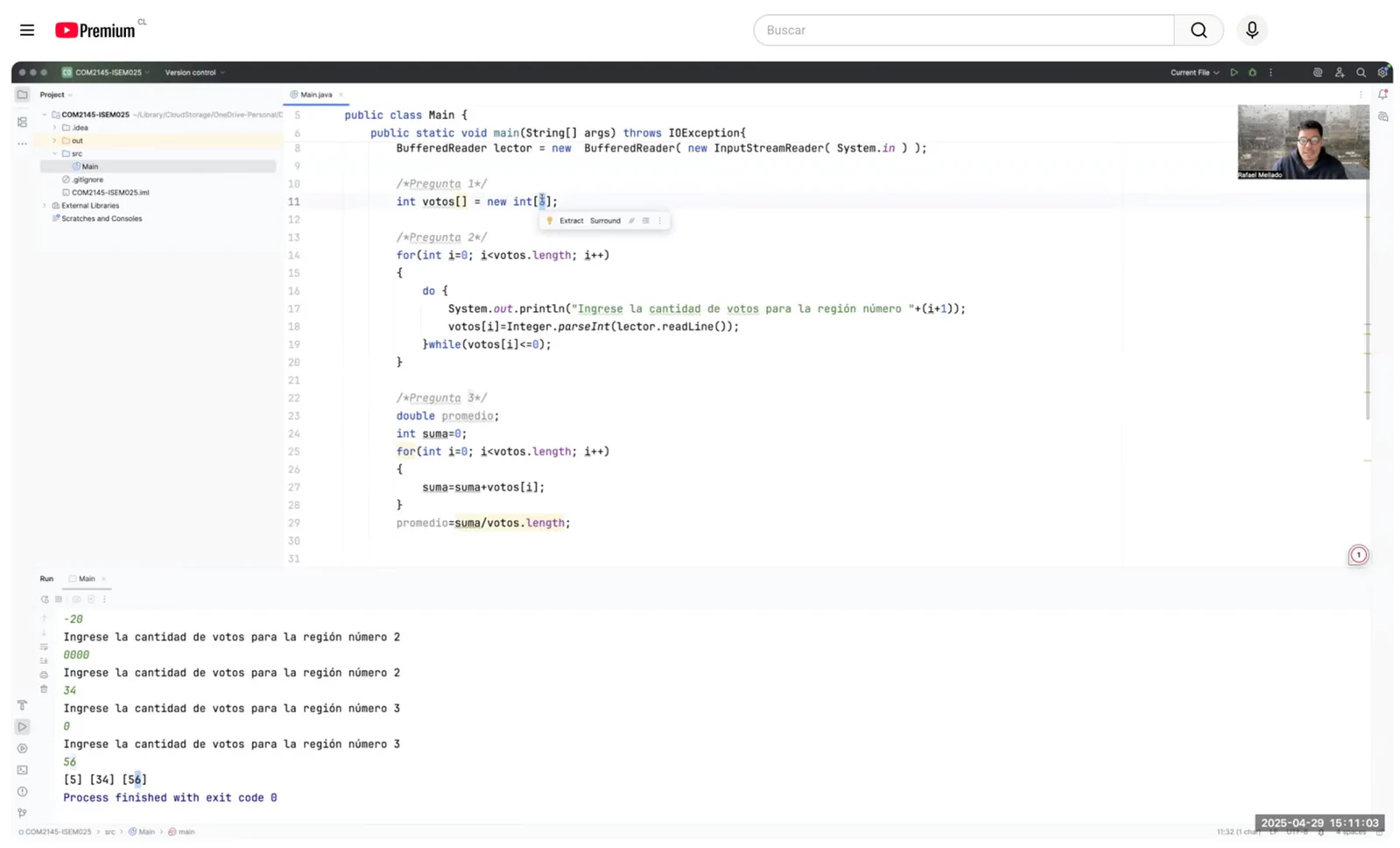
A screenshot of an explanatory video of the exercises can be found in the guide.

The two conditions differed only in terms of support resources. The control group used a video created to review the content of the exercises, which explained the underlying concepts without answering the guide’s questions; thus, responding still required analysis on the part of the student (Figure 3).

**Figure 4:**
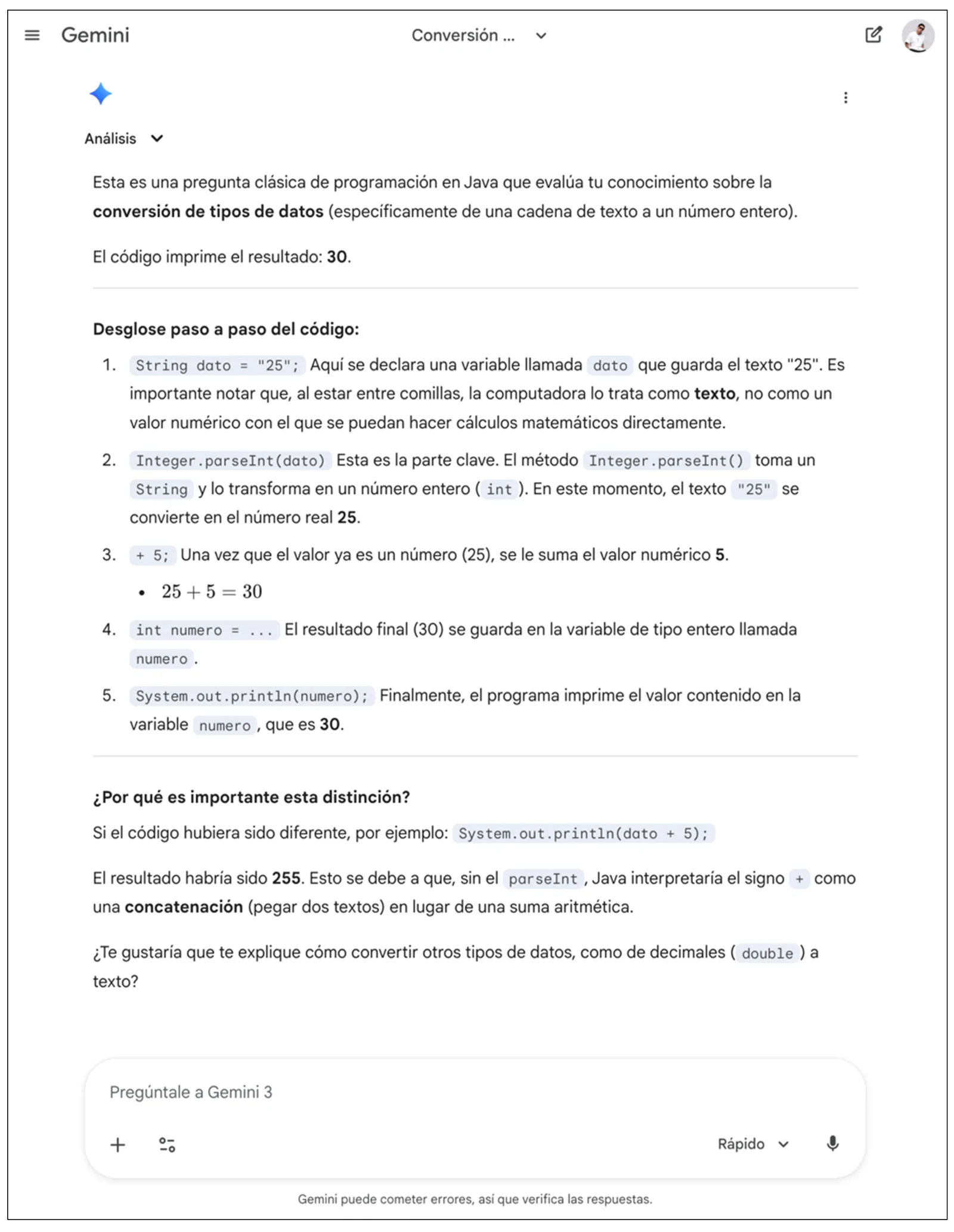
Example of student interaction with Google Gemini.

The experimental group was instructed to consult Google Gemini and produce analytical responses based on its output, since copying the generated result was insufficient for the purpose of the activity; this instruction was intended to foster metacognitive awareness and limit overreliance on the tool’s suggestions. Figure 4 shows an example of student interaction, in which a text string is converted into an integer value.

### 3.4. Instrument

For data collection, academic performance evaluations and technical knowledge assessments were excluded, focusing the study exclusively on user perceptions and technology adoption. The primary instrument consisted of a self-administered questionnaire that integrated constructs from the Technology Acceptance Model (TAM) and the Intrinsic Motivation Inventory (IMI) and was designed to measure changes in perceptions before (pre-test) and after (post-test) the intervention.

The psychometric instrument comprises four fundamental dimensions, each evaluated on a 5-point Likert scale ranging from strongly disagree to strongly agree:

- Perceived Ease of Use evaluates the effort required to interact with the support tool and determines whether the interaction is clear and free of excessive mental load.
- Behavioral Intention measures the student’s behavioral predisposition to continue using the assigned technology in future learning activities or autonomous study.
- Value/Usefulness assesses the extent to which students internalize the pedagogical activity as structurally valuable and beneficial for their cognitive development.
- Perceived Enjoyment captures the hedonic component and the experiential satisfaction reported during task resolution.

The theoretical framework and empirical design are related through the restricted operationalization of cognitive friction. Cognitive friction and cognitive load are not quantified using dedicated psychometric scales, such as the NASA-TLX, or objective performance metrics, such as error rates. Cognitive friction is operationalized exclusively as a theoretical proxy inferred from the Perceived Ease of Use construct, so that the barriers experienced by students are measured through self-reported perceptual data alone.

The practical stimulus was identical for both conditions because the Java exercise guide did not vary between groups. This standardization removes differences in the complexity or design of the exercises as a source of variation observed in the constructs, given that the conditions differ only in the support resource. Table 1 details the operationalization of constructs and items used.

The psychometric properties of the adapted TAM and IMI instruments were thoroughly evaluated to ensure reliability and validity within this specific educational context. To establish content validity, the original measurement scales were translated into Spanish and terminologically contextualized for algorithmic programming with GenAI. The adapted items were reviewed by domain experts to confirm semantic equivalence and pedagogical relevance prior to deployment. Furthermore, internal consistency reliability was assessed for all dimensions using Cronbach’s alpha. The reliability coefficients demonstrated high internal consistency across both pre-test and post-test measurements for the constructs of Perceived Enjoyment ($\alpha_{pre}=0.82,\;\alpha_{post}=0.84$), Perceived Ease of Use ($\alpha_{pre}=0.85,\;\alpha_{post}=0.87$), Behavioral Intention ($\alpha_{pre}=0.89,\;\alpha_{post}=0.91$), and Value/Usefulness ($\alpha_{pre}=0.87,\;\alpha_{post}=0.88$).

### 3.5. Data Analysis

For the Wilcoxon signed-rank tests, the V statistic is computed over pairs with a non-zero difference, so that its magnitude is not directly comparable with the number of participants. The RCI determines whether an individual’s score change exceeds the measurement-error threshold (Jacobson & Truax, 1991). The calculation requires the Standard Error of Measurement (SEM) and the Standard Error of the Difference ($S_{diff}$), defined as:

(1) $$SEM=s_{1}\sqrt{1-r_{xx}}$$

(2) $$S_{diff}=\sqrt{2\cdot SEM^{2}}$$

(3) $$RCI=\frac{X_{post}-X_{pre}}{S_{diff}}$$

where $s_{1}$ is the baseline standard deviation, and $r_{xx}$ represents the reliability coefficient. Given the single pre-test administration, internal consistency (Cronbach’s alpha) was utilized as a proxy for test–retest reliability. An RCI≥1.96 indicates a statistically reliable positive change at the 95% confidence level.

## 4. Results

### 4.1. Pre-Test and Post-Test Changes by Group

Table 2 reports the average scores by group and dimension, showing that both groups began from comparable levels in Enjoyment and Behavioral Intention and differed more markedly in Perceived Ease of Use and Value/Usefulness, whereas at post-test the widest separations appeared in Enjoyment, Perceived Ease of Use, and Value/Usefulness, with an average change in Behavioral Intention of nearly identical magnitude in the two groups.

**Figure 5:**
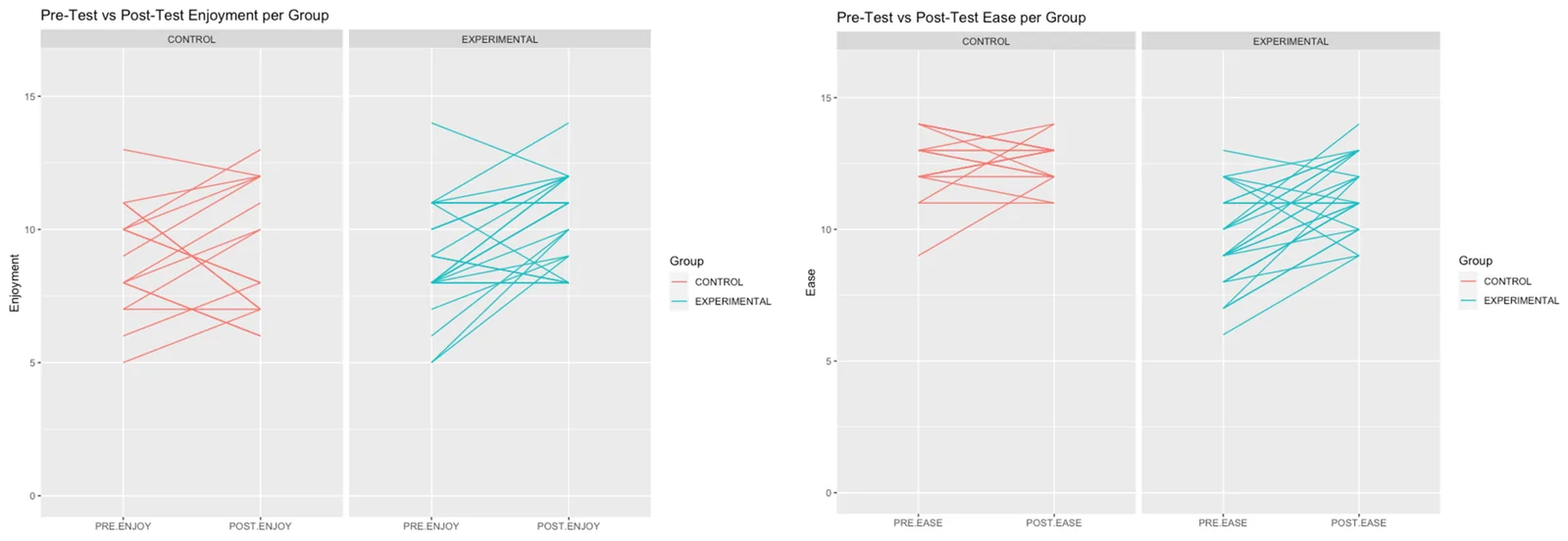

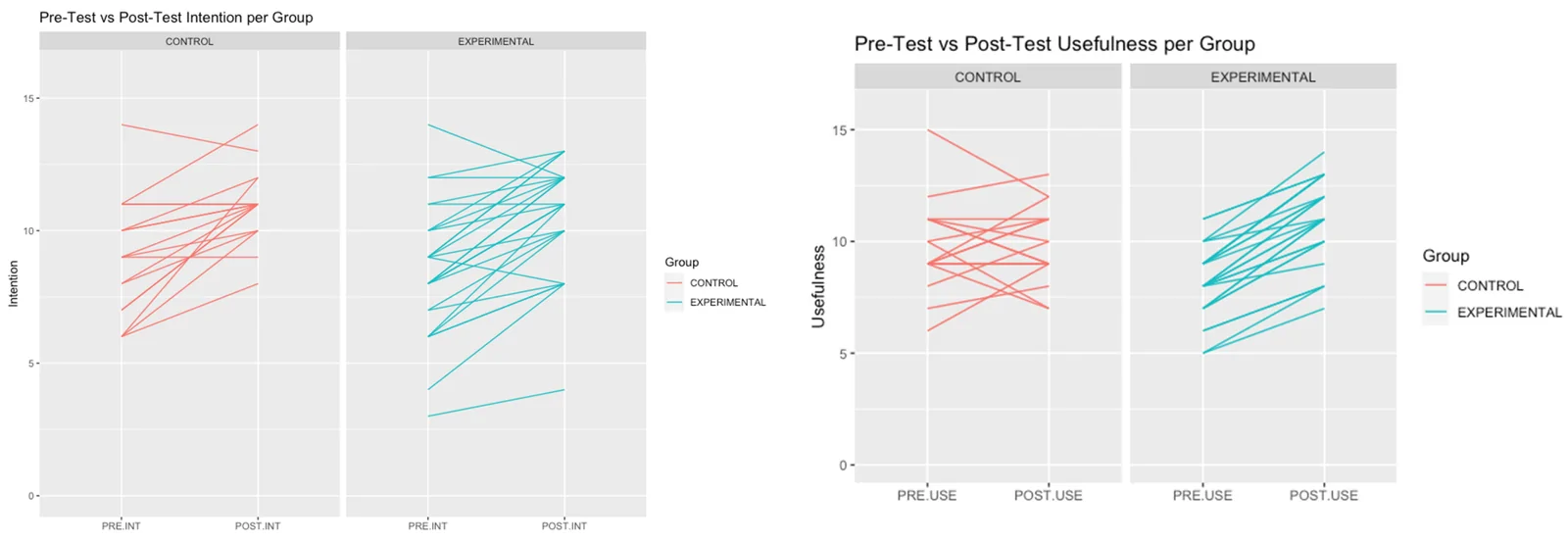
Evolution of pre- and post-test scores for the dimensions of Enjoyment, Ease of Use, Behavioral Intention, and Value/Usefulness.

Figure 5 shows the individual trajectories, in which progression in Enjoyment was comparable across groups, whereas in Perceived Ease of Use and Value/Usefulness the experimental group followed a consistent upward trend that the control group did not exhibit. Both groups tended to increase in Behavioral Intention, although the experimental participants started from a wider and lower range of initial scores.

In the control group, following verification of the distributional assumptions, a significant pre-post difference was obtained only in Behavioral Intention (paired-samples *t*-test, t(56) = 4.489, *p* < 0.001, d = 0.59, 95% CI [0.31, 0.88]). The remaining dimensions provided no evidence that the descriptive changes were statistically significant (Enjoyment, Wilcoxon signed-rank test, V = 58.5, *p* = 0.636, r = 0.06, 95% CI [−0.20, 0.32]; Perceived Ease of Use, V = 47, *p* = 0.941, r = 0.01, 95% CI [−0.25, 0.27]; Value/Usefulness, t(56) = 0.243, *p* = 0.811, d = 0.03, 95% CI [−0.23, 0.29]).

In the experimental group, under the same procedure, significant pre-post differences were obtained in all four dimensions (Enjoyment, V = 26, *p* = 0.002, r = 0.40, 95% CI [0.16, 0.59]; Perceived Ease of Use, t(59) = 4.906, *p* < 0.001, d = 0.63, 95% CI [0.36, 0.91]; Behavioral Intention, V = 14, *p* < 0.001, r = 0.52, 95% CI [0.30, 0.68]; Value/Usefulness, t(59) = 14.99, *p* < 0.001, d = 1.94, 95% CI [1.51, 2.36]). Effect sizes were expressed as post-test minus pre-test, so that positive values denoted an increase.

### 4.2. Individual Results

**Figure 6:**
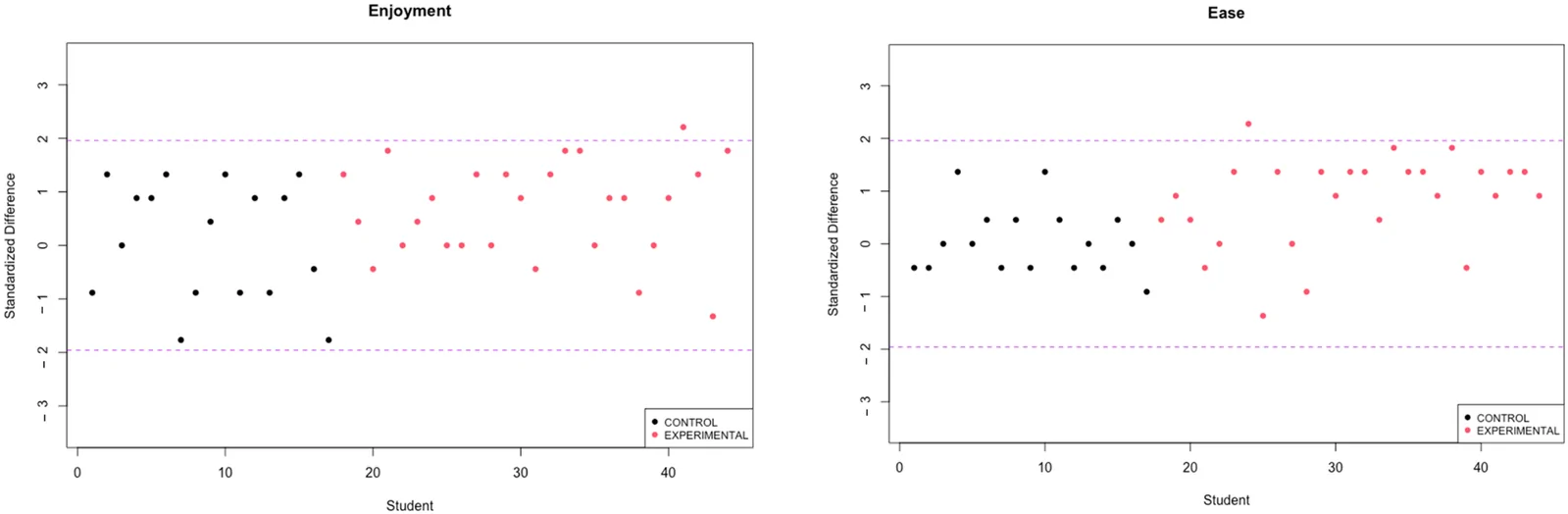

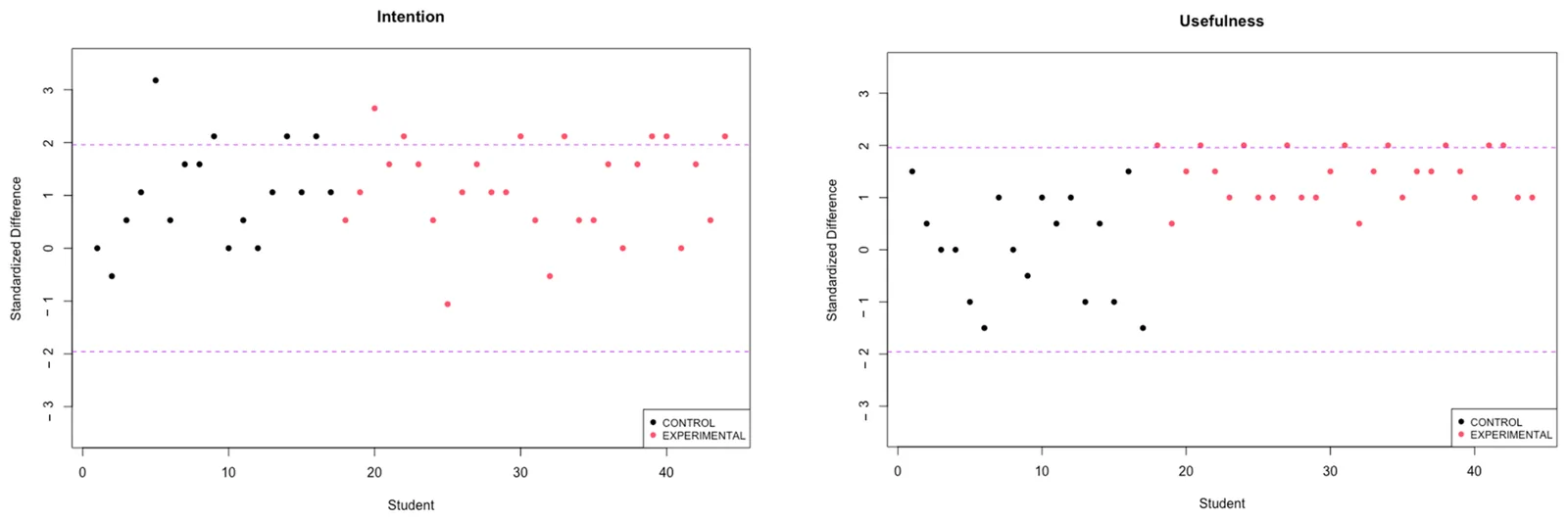
RCI per student for the dimensions of Enjoyment, Ease of Use, Behavioral Intention, and Value/Usefulness.

Figure 6 illustrates the RCI calculations per student for each framework dimension. The analysis of individual cases reveals similar patterns in Enjoyment and Ease of Use, with the majority of students reporting positive changes; however, only one student from the Experimental group exceeds the +1.96 threshold in both cases. This situation differs from the observations in the Intention dimension, where a greater number of variations exceed the +1.96 threshold, including students from both categories. It should also be noted that in Intention, most subjects exhibit positive changes following the intervention. The analysis of the Value/Usefulness dimension indicates that all cases recording positive variations exceeding the +1.96 threshold belong exclusively to the Experimental group. However, despite the statistically significant group-level changes, only a small fraction of individuals across all constructs exceeded the stringent ±1.96 threshold, indicating that the intervention produced a generalized systemic shift rather than extreme individual outliers.

## 5. Discussion

The primary objective of this study was to analyze the adoption dynamics of Generative AI among non-technical students engaged in high-load programming tasks. Contrary to the expectation that operational ease dictates immediate acceptance, the findings suggest a trajectory marked by initial cognitive friction followed by significant gains in intrinsic motivation, pedagogical value, and behavioral intention.

### 5.1. Change in Technology Acceptance over the Intervention

The initial divergence between the conditions challenges utilitarian accounts of adoption in education. At the outset, the control group rated the instructional videos as easier and more useful than the experimental group rated its experience with GenAI, a pattern consistent with the observation of Sprenger and Schwaninger (2021) that passive resources are accepted rapidly because of their simplicity, whereas highly interactive tools meet initial resistance and are judged less valuable until adequately contextualized.

The lower baseline in the experimental condition is consistent with recent studies on GenAI. Videos present a determinate format, while GenAI requires specific technical skills and ethical awareness that generate uncertainty among non-expert students (Vhatkar et al., 2024), and Andrade-Girón et al. (2024) note that the successful integration of these tools depends on closing gaps in user familiarity.

The change observed in the experimental condition after the intervention indicates that the participants were able to overcome the initial difficulty. In theoretical terms, the trajectory suggests that under high cognitive friction, ease of use does not by itself produce adoption and requires the concurrence of perceived pedagogical value, a condition that Shata and Hartley (2025) formulate when they observe that operational simplicity is insufficient for academic adoption in the absence of tangible benefits. The reduction in initial difficulty coincides with gains in technical proficiency and enjoyment, which supports Musa et al. (2024) that adoption models should incorporate hedonic factors when the technology is disruptive.

### 5.2. Navigating Cognitive Friction and the Role of Intrinsic Motivation

The affective and intentional dimensions revealed qualitative differences between the conditions. The control group maintained stable levels of enjoyment and pedagogical value throughout the study, whereas the experimental group registered significant increases in Value/Usefulness and Enjoyment. Vhatkar et al. (2024) attribute this displacement to the capacity of GenAI to generate personalized environments that move the experience from passive reception toward active construction, a shift that appears necessary to sustain motivation during programming tasks with a high cognitive load.

The intra-subject rise in value/usefulness is consistent with the interpretation that, for non-expert users, the utility of GenAI is not presupposed but emerges once the initial friction has been traversed. Within the experimental condition, enjoyment and intrinsic pedagogical value operate as the mechanisms that sustain behavioral commitment, which the increase in Perceived Ease of Use alone does not account for. Andrade-Girón et al. (2024) describe a comparable dynamic in which the confirmation of time savings and improved learning management reinforces enjoyment.

The association between perceived ease of use and use documented by Or (2024) acquires a distinct form in this setting, since ease is not a stable attribute of the tool but a proficiency acquired over the period of interaction, and behavioral intention follows the increase in instrumental capability rather than preceding it.

A methodological observation follows from the contrast between group-level results and individual RCI outcomes. The inferential tests yielded significant differences, while only a small subset of students exceeded the +1.96 threshold, a divergence attributable to the different sensitivities of the two metrics. Group-level statistics respond to systematic unidirectional displacements, which is what the intervention produced, namely a consistent moderate increase across most of the experimental sample that raises the mean, while the variance remains low. The RCI is a conservative metric designed to separate individual change from measurement error; thus, the limited number of threshold crossings indicates that five weeks are insufficient to generate individual changes large enough to exceed a conservative error margin, even when the direction of change is consistent.

### 5.3. Task Complexity and Cognitive Load Interactions with Adoption

The complexity of the task interacts with the modality of support, consistent with the Cognitive Load Theory. Acceptance was measured after the complete exercise guide, but the progression from a linear procedure, such as conditional structures, toward the abstract handling of data structures allows the role of GenAI as cognitive scaffolding to be examined.

Passive resources are well received in simpler initial tasks because their operational friction is low, and they become insufficient to sustain engagement as intrinsic cognitive load increases toward the end of the activity. The higher post-intervention acceptance of GenAI in the experimental condition suggests that its instructional contribution lies in providing a personalized environment that supports logical abstraction (Vhatkar et al., 2024), since problem decomposition reduces extraneous load and allows attention to remain on programming logic. Jensen et al. (2025) relate a favorable view of these tools to their capacity to support personalized learning in tasks that would otherwise require intensive supervision.

The relative weights of the constructs shift as tasks move from low to high complexity, and in this study, increases in Perceived Ease of Use and Enjoyment accompanied the reported capacity to manage a growing cognitive load, which is consistent with Abdalla’s (2024) observation that awareness of the capabilities of the tool and enjoyment of its features condition the adoption of ChatGPT.

### 5.4. Pedagogical Relevance for Digital Literacy in Non-Technology Students

These findings indicate that GenAI can increase students’ willingness to engage in complex technical tasks. Teaching programming in the social sciences encounters affective as well as cognitive obstacles, and the perceptual data obtained here suggest that the tool offers motivational support to students without confidence in advanced mathematics, sustaining their engagement with abstract constructs such as arrays. Bray (2024) describes the same displacement toward natural-language programming, in which non-expert students avoid syntactic memorization and concentrate on problem logic.

The reduction in initial technical barriers moves the pedagogical focus from mechanical execution to critical oversight, which Deroncele-Acosta et al. (2025) associate with the development of higher-order skills. The corresponding risk is that the facility of adoption produces an illusion of competence, and Beale (2025) accordingly locates digital literacy in verification skills such as auditing generated code for hallucinations, without which the tool displaces analytical reasoning rather than supporting it (Vieira & Mesquita, 2025).

A hybrid curricular design remains a pertinent response for this student population. The association between behavioral intention and task difficulty suggests that non-STEM students regard these systems as a cognitive aid for a data-intensive labor market, and Machado et al. (Machado et al., 2025) situate the future of university instruction in an ethical partnership with these tools that preserves disciplinary integrity.

### 5.5. Limitations

The interpretation of these findings is bounded by several methodological constraints, since the sample consisted of Accounting and Auditing students in a single Latin American institution and, although Andrade-Girón et al. (2024) argue for the need for evidence from this region, the specialized curricular profile restricts transfer to other fields. The data were self-reported, which admits social desirability bias, and no objective measures of academic performance or skill acquisition were collected. Therefore, the findings describe perceived pedagogical value and behavioral intention rather than learning effectiveness. Triangulation with examination results and recorded usage data, as Or (2024) recommends, is required before the observed motivational gains can be related to cognitive outcomes.

The inferential design further restricts the scope of these conclusions, given that the contrasts reported here are within-group comparisons between the pre- and post-tests in each condition and that no direct between-group test of the change was conducted, so that the difference between a significant result in one condition and a non-significant result in the other does not, by itself, establish a difference between conditions. The two conditions were moreover not equivalent at baseline in Perceived Ease of Use and differed descriptively in Value/Usefulness, which means that part of the change observed in the experimental condition may reflect its lower starting point.

The immediacy of the measurement admits a novelty effect, as Sprenger and Schwaninger (2021) reported that interest in disruptive technologies declines once their use becomes routine. Therefore, longitudinal designs spanning a full academic year are required to determine whether the perceived value is maintained.

Cognitive load was not measured using a validated instrument such as the NASA-TLX, since cognitive friction was inferred from the Perceived Ease of Use construct, and task complexity was established a priori from the learning objectives, which leaves the mental effort actually incurred unquantified.

The study evaluated Value/Usefulness, Ease of Use, and Enjoyment and excluded exogenous variables such as social influence, institutional trust, and subjective norms, which Shata and Hartley (2025) identified as conditions of GenAI adoption and which Vhatkar et al. (2024) listed among the pending questions in the literature. The framework was deliberately hybrid rather than a classical TAM specification, and the operational Perceived Usefulness construct was replaced by the Value/Usefulness subscale of the IMI, which captures intrinsic pedagogical value. Studies seeking to validate classical adoption models in GenAI contexts should reincorporate operational measures to contrast utilitarian efficiency with intrinsic motivation.

## 6. Conclusions

This study examined the adoption of Generative Artificial Intelligence among university students without technical preparation who were engaged in Java programming tasks of increasing complexity within a framework that combines technology acceptance constructs with intrinsic motivation constructs. The two support modalities produced different perceptual trajectories, since the instructional videos afforded immediate, stable ease that did not change over the intervention period, whereas GenAI followed an adoption curve that began with initial difficulty and subsequently registered significant within-group increases in all four dimensions measured. In the control condition, the only significant change occurred in the Behavioral Intention.

The reduction in the perceived complexity barrier associated with GenAI was not immediate, since interaction with the tool initially demanded cognitive effort in the form of prompt literacy that depressed perceived ease of use. The perceptual data indicate that appreciation of the pedagogical value of the tool and of its scaffolding capacity becomes apparent while students work with more abstract content such as arrays, and that enjoyment increased significantly only in the experimental condition.

The theoretical contribution of this study lies in questioning the static treatment of the Technology Acceptance Model. Unlike conventional software, in which initial ease of use is a precondition for adoption, the trajectory documented here indicates that behavioral intention toward GenAI is resilient to initial cognitive friction as long as the tool provides instrumental scaffolding during tasks with a high cognitive load. At the practical level, this implies that curricular design in the business sciences should not treat the initial technical difficulty of these tools as an obstacle to be avoided, and should instead incorporate deliberate induction phases that move students from perceived difficulty toward technical competence, which is the basis of the digital self-efficacy required of future auditors and analysts.

These conclusions describe the perceptions and behavioral intentions reported by a specific group of Latin American students under a within-group design, and they do not extend to academic performance or the acquisition of cognitive skills, neither of which was measured. Determining whether the acceptance and enjoyment recorded here translate into durable programming competence requires objective performance data and longitudinal follow-up beyond the initial novelty period.

## Figures and Tables

**Table 1:** Questions and constructs of the TAM instrument.

Construct	Question/Statement
Perceived Enjoyment	I will enjoy performing the activity. I think this will be a boring activity. The experience of the activity will be pleasant and enjoyable.
Value/Usefulness	I believe this activity could be of some value to me. I think that doing this activity is useful for learning the material. I believe doing this activity could be beneficial to me.
Perceived Ease of Use	Interaction with the tool will be easy to perform. I find that the activity will be easy to use. Interaction with the activity will be clear and understandable.
Behavioral Intention	I would plan to use it in the future for reviewing or studying. I expect to continue using it in the future. I intend to continue using it during the semester.

**Table 2:** Average score by group and by dimension.

Average by Group	Enjoyment			Value/Usefulness			Perceived Ease of Use			Behavioral Intention		
	Pre-Test	Post-Test	Diff	Pre-Test	Post-Test	Diff	Pre-Test	Post-Test	Diff	Pre-Test	Post-Test	Diff
Control	8.9	9.2	0.3	9.8	9.9	0.1	12.5	12.6	0.1	8.9	10.9	2.0
Experimental	8.9	10.4	1.5	8.2	11.0	2.8	9.6	11.4	1.8	8.4	10.6	2.2

## Data Availability

The data presented in this study are available on request from the corresponding author. The data are not publicly available due to privacy restrictions.
